# Dietary Cerium (Ammonium Ceric Nitrate) Promoted the Growth, Intestinal Digestive Enzyme Activity, and Positive Modulation of Intestinal Microbiota of Largemouth Bass (*Micropterus salmoides*)

**DOI:** 10.3390/ani16030506

**Published:** 2026-02-05

**Authors:** Yugui Zhang, Yunfeng Chen, Kaihui Xu, Xiaoqin Li, Xiangjun Leng

**Affiliations:** 1 National Demonstration Center for Experimental Fisheries Science Education, Shanghai Ocean University, Shanghai 201306, China; z09vad@163.com (Y.Z.); chenyf256893@163.com (Y.C.); x19990707kh@163.com (K.X.); 2 Research Center for Fish Nutrition and Environmental Ecology, Ministry of Agriculture and Rural Affairs, Shanghai Ocean University, Shanghai 201306, China

**Keywords:** *Micropterus salmoides*, dietary cerium, appropriate inclusion, positive modulation

## Abstract

This study investigated the dietary effects of cerium (ammonium ceric nitrate, Ce (NH_4_)_2_(NO_3_)_6_) on the growth, serum antioxidant, intestinal digestive enzyme activity, tissue morphology and microbiota of *Micropterus salmoides*. Seven diets were designed with cerium supplementation of 0 (CON), 10 (Ce10), 20 (Ce20), 40 (Ce40), 60 (Ce60), 80 (Ce80) and 120 mg/kg (Ce120), respectively. Largemouth bass juveniles (initial weight of 16.89 ± 0.04 g) were fed with the above diets for 56 days. Compared with the control group, the weight gain of the Ce40 group increased by 14.4% and the feed conversion ratio decreased by 0.13 (*p* < 0.05). The Ce60, Ce80 and Ce120 groups showed significantly higher superoxide dismutase activity and lower malondialdehyde concentration compared with the control group (*p* < 0.05). Protease activity in the Ce20 and Ce40 groups and amylase activity in the Ce40 group were markedly elevated relative to the control group (*p* < 0.05). The proportion of Firmicutes was increased and the proportion of Proteobacteria was decreased by the addition of 10 mg/kg and 40 mg/kg Ce (Ce10 and Ce40 groups). In summary, dietary cerium supplementation can promote the growth, intestinal digestive enzyme activity, and positive modulation of the intestinal microbial flora of juvenile *Micropterus salmoides*. Based on the second-order polynomial regression analysis of WG or the FCR, the appropriate inclusion level of dietary cerium for juvenile largemouth bass was estimated to be 57.9 and 60.0 mg/kg, respectively.

## Simple Summary

Largemouth bass (*Micropterus salmoides*) is the most important carnivorous fish cultured in China. With the development of high-density farming, the potential risks faced are continuing to grow, and exploring green functional additives is of great significance for the healthy development of the largemouth bass farming industry. The present study investigated the impacts of supplementing cerium as the form of ammonium ceric nitrate (Ce (NH_4_)_2_(NO_3_)_6_) in feed on the growth, serum antioxidant and immune function, intestinal digestive enzyme activity, tissue morphology and microbiota of largemouth bass. The results indicated that the addition of 40 mg/kg dietary cerium significantly increased weight gain and decreased the feed conversation ratio, and the higher inclusion did not further increase the weight gain and decrease the feed conversation ratio. In summary, dietary cerium supplementation can promote the growth, intestinal digestive enzyme activity, and positive modulation of the intestinal microbial flora of juvenile largemouth bass. Based on the second-order polynomial regression analysis of WG or the FCR, the appropriate inclusion level of dietary cerium for juvenile largemouth bass was estimated to be 57.9, and 60.0 mg/kg, respectively. The findings will direct the application of rare earth additives in aquatic feeds.

## 1. Introduction

Rare earth elements refer to 17 metallic elements including the lanthanide series (15 elements with atomic number ranging from 57 to 71) as well as scandium (Sc) and yttrium (Y). In livestock, rare earth elements have shown multiple functions such as regulating enzyme activity, modulating immune function, promoting nutrients digestion and absorption, and exhibiting antibacterial properties [1]. However, the research of rare earth elements in aquatic animals is relatively limited. In turbot (*Scophthalmus maximus*), dietary rare earth nitrate (240 mg/kg) significantly improved survival and weight gain [2]. Dietary supplementation of 0.2 g/kg of rare earth elements can effectively enhance the weight gain and increase the carcass weight of golden pompano (*Trachinotus ovatus*) [3]. The inclusion of 200–300 mg/kg rare earth nitrate also enhanced the weight gain and intestinal trypsin, chymotrypsin, and lipase activities of common carp (*Cyprinus carpio*), but excessive addition inhibited the activities of these enzymes [4].

Cerium is the most abundant rare earth element in the earth’s crust, and it has two valence states, trivalent and tetravalent. The tetravalent form of cerium ions exhibits stronger oxidizing properties. It has been reported that the addition of cerium oxide in feed significantly reduced serum malondialdehyde level of laying hens, thereby enhancing the antioxidant stability and positively influencing the shelf life of eggs [5]. In Gibel carp (*Carassius auratus gibelio*), dietary supplementation of rare earth-chitosan chelate (containing approximately 3% cerium and 5% lanthanum) significantly improved growth performance and non-specific immune capacity [6]. Adding 200 mg/kg of cerium–chitosan complex to diet significantly reduced cadmium accumulation in *Scophthalmus maximus* [7]. Qin et al. [8] once reported that the supplementation of 0.8 mg/kg of nano-cerium oxide in feed enhanced the stress resistance and immune capacity of Chinese mitten crabs (*Eriocheir sinensis*).

The largemouth bass is the most important carnivorous fish cultured in China, and its aquaculture production reached 938,509 tons in 2024 [9]. With the development of high-density farming, the potential risks are continuing to grow, and exploring green functional additives is of great significance for the healthy development of the largemouth bass farming industry. Considering the beneficial effects of dietary cerium in other animals and the lack of relevant reports on dietary cerium supplementation in largemouth bass, the present study investigated the effects of dietary cerium addition (ammonium ceric nitrate, Ce (NH_4_)_2_(NO_3_)_6_) on the growth, serum antioxidant and immune function, intestinal digestive enzyme activity, intestinal morphology and microbiota of largemouth bass. The findings will direct the application of rare earth additives in aquatic feeds.

## 2. Materials and Methods

### 2.1. Experimental Design and Diets

Ammonium ceric nitrate (purity of 99% and cerium content of 25.56%, Macklin Biochemical Technology Co., Ltd., Shanghai, China) was added to the basal diet (49% crude protein, 13% crude fat) at concentrations of 39.13, 78.25, 156.49, 234.74, 312.99 and 469.48 mg/kg, corresponding to dietary cerium contents of 10 (Ce10), 20 (Ce20), 40 (Ce40), 60 (Ce60), 80 (Ce80), and 120 (Ce120) mg/kg, respectively (Table 1). The feed ingredients were ultra-fine ground and sieved through a 60-mesh screen. After the solid ingredients were ground through the 60 mesh, all ingredients were mixed evenly. A single-screw extruder (LX-75 extruder, Longxiang Food Machinery Factory, Xingtai City, China) was used to produce slow-sinking pellets with a particle size of 2 mm. The pelleting temperature was 85 ± 5 °C. The feed was dried to a moisture content of approximately 100 g/kg and stored at room temperature in sealed containers.

### 2.2. Experimental Animals and Feeding Management

After being temporarily stocked in indoor cement pools for two weeks, 630 healthy fish from China Daming Lake Aquaculture Farm (average weight of 16.89 ± 0.04 g) were selected for the feeding trial. The fish were fed in cages (1.5 × 1.2 × 1.0 m) hung in cement pools (5.0 × 3.0 × 1.2 m) without direct sunshine at the Shanghai Ocean University Binghai Aquaculture Base. The experiment included seven treatments with three cages per treatment and 30 per cage. During the feeding period of 56 days, the feed was provided twice daily at 3–5% of the fish’s body weight (8:30, 16:30), which was adjusted based on feeding behavior to maintain consistent feeding rates across cages. The feces waste was cleaned twice weekly to maintain water quality, and 1/10 of the water volume was renewed every week. During the feeding period, water temperature was 23–30 °C with pH 6.5–7.3, dissolved oxygen > 4 mg/L, salinity 0.5–1.0‰, nitrite < 0.1 mg/L, and ammonia nitrogen 0.1–0.2 mg/L.

### 2.3. Sampling

All sampling procedures (weighing, blood draw, dissection) were performed under anesthesia with buffered MS-222 (100 mg/L). All handling was standardized and completed within 2 min to minimize acute stress. When the fish reached an anesthetized state characterized by complete cessation of opercular movement without response to mechanical stimulus, sampling was performed immediately.

All fish from each cage were carefully transferred into a pre-weighed container with a standardized volume of water; then, the total weight of fish was immediately recorded when the electronic scale reading stabilized. This identical “wet-weighing” procedure was applied consistently at both the beginning (Day 0) and the end (Day 56) of the feeding trial.

A total of 10 fish per cage were randomly selected for subsequent sampling. (1) A total of 3 fish per cage were used for whole-body composition analysis (*n* = 3 fish per cage; total 9/treatment). (2) Another 3 fish per cage were selected for measurements of length/weight followed by blood collection and dissection (*n* = 3 fish per cage; total 9/treatment). Specifically, blood was drawn from the caudal vein, then centrifuged at 3000 rpm for 10 min, and the serum was stored at −80 °C for enzyme activity assays. After the blooding, the fish were dissected immediately, and the viscera and liver were sampled and weighed. Under sterile conditions, the intestine was separated, and a 1 cm segment of the foregut was fixed with Bouin’s solution for histological examination, while the remaining portion was used for intestinal enzyme activity measurements. (3) Another 4 fish per cage were dissected under sterile conditions, and 1 cm of the hindgut was separated and stored in liquid nitrogen for detection of intestinal microbiota (*n* = 4 fish per cage with 2 fish pooled as 1 sample; total 6/treatment) only in the selected treatments (CON, Ce10, Ce40, Ce120).

### 2.4. Measurement Indicators and Methods

#### 2.4.1. Growth, Body Morphometric Indices and Nutrient Retention

Weight gain (WG, %) = 100 × [final weight(g) − initial weight (g)]/initial weight (g)

Feed conversation ratio (FCR) = feed intake (g)/[final weight (g) − initial weight(g)]

Survival rate (SR, %) = 100 × final number of fish/initial number of fish

Condition factor (CF, g/cm^3^) = body weight (g)/body length (cm)^3^

Viscerosomatic index (VSI, %) = 100 ×visceral weight (g)/body weight (g)

Hepatosomatic index (HSI, %) = 100 × liver weight (g)/body weight (g)

Protein retention (PR, %) = 100 × (W_2_ × W_2_CP − W_1_ × W_1_CP)/(FI × FCP);

Lipid retention (LR, %) = 100 × (W_2_ × W_2_CL − W_1_ × W_1_CL)/(FI × FCL).

where W_2_ is the final fish weight (g); W_1_ is the initial fish weight (g); W_2_CP is the crude protein content of the final fish (%); W_1_CP is the crude protein content of the initial fish (%); W_2_CL is the crude lipid content of the final fish (%); W_1_CL is the crude lipid content of the initial fish (%); FI is the feed intake (g); FCP is the crude protein content of the feed (%); and FCL is the crude lipid content of the feed (%).

#### 2.4.2. Whole-Body Composition

Following the method described by AOAC [11], the moisture, ash, and crude protein content of feed and whole fish were analyzed. Moisture content was determined using the drying method at 105 °C. Crude protein content was determined using the Kjeldahl nitrogen determination method (Kjeltec 2300, Foss, Tecator AB, Höganäs, Sweden), and crude ash content was determined using the 550 °C combustion method with muffle furnace (SXL-1008, Jinhong Experimental Equipment Co., Ltd., Shanghai, China). In addition, crude lipid was determined using the chloroform–methanol method [12].

#### 2.4.3. Serum Biochemical Analysis

Serum biochemical parameters were determined using the biochemical kits provided by Shunshi Biotechnology Co., Ltd. (Shanghai, China). The parameters included superoxide dismutase (SOD, xanthine oxidase method), catalase (CAT, colorimetric method), total antioxidant capacity (T-AOC, colorimetric method), malondialdehyde (MDA, colorimetric method of TBA), total protein (TP, colorimetric method), alkaline phosphatase (ALP, colorimetric method), acid phosphatase (ACP, colorimetric method), lysozyme (LZM, turbidimetric method), total nitric oxide synthetase (T-NOS, colorimetric method), and complement 3 (C3, immunoturbidimetric method).

#### 2.4.4. Intestinal Digestive Enzyme Activities

The intestinal trypsin and amylase activities were determined using kits produced by Jiancheng Bioengineering Institute (Nanjing, China). The pre-treatments were as follows: the frozen foregut samples were thawed at 4 °C, then homogenized with 9 times the volume of 4 °C saline (8.6 g/kg NaCl), centrifuged at 1500 *g* for 15 min at 4 °C. Finally, the supernatant was separated, and digestive enzyme activities were determined within 24 h.

#### 2.4.5. Intestinal Tissue Morphology

After a series of dehydration with different concentrations of ethanol, the foregut tissues were transparentized with xylene, then embedded in paraffin wax, and sections were cut with a thickness of 5 μm using a sectioning machine (Leika RM 2235, Wetzlar, Germany), stained with hematoxylin–eosin (HE). The intestine tissues were observed and photographed using a microscope (Nikon YS100 Photomicrography System, Tokyo, Japan), and the height and width of the intestinal villi and the thickness of the muscularis propria were recorded (Image J, v1.8.0).

#### 2.4.6. Intestinal Microbiota

The intestinal microbial samples were processed for DNA extraction, followed by PCR-based amplification and high-throughput sequencing on the Illumina MiSeq Platform. The V3-V4 regions of the 16S rRNA gene were targeted for amplification with V338F (5′-ACTCCTACGGGGAGGCAGCAG-3′) and V806R (5′-GGACTACHVGGGTWTCTAAT-3′) primers. The number of 16S rRNA gene sequences from each sample was rarefied to 20,000, which yielded an average Good’s coverage of 99.09%. OTU clustering analysis was performed via the Shanghai Majorbio Cloud Online Platform (https://cloud.majorbio.com) to characterize microbial community composition and species abundance across phylum and genus levels. The entire experiment was completed in Shanghai Majorbio Biopharmaceutical Technology Co., Ltd. (Shanghai, China).

### 2.5. Statistical Analysis

The experimental data were expressed as mean ± standard deviation (SD). All data were tested for normality and homogeneity of variance and then analyzed by one-way analysis of variance (ANOVA) with SPSS 26.0 analysis software. Multiple comparisons among treatments were performed using Duncan’s method with a significance level of *p* < 0.05 [13,14]. The second-order polynomial regression model was used to test the appropriate inclusion level of cerium based on WG or the FCR.

The experimental units for growth performance indicators (Table 2) are 3 cages per treatment, with a total *n* of 3/treatment. The experimental units for body morphometric indices (Table 3), nutrient retention and whole-body composition (Table 4), serum biochemical indicators (Table 5), intestinal digestive enzyme activities (Table 6) and tissue morphology indicators (Table 7) are 3 fish per cage, with a total *n* of 9/treatment. The experimental units for intestinal microbiota indicators are 4 fish per cage (2 fish pooled as 1 sample), with a total *n* of 6/treatment (Table 8).

## 3. Results

### 3.1. Growth Performance

**Figure 1:**
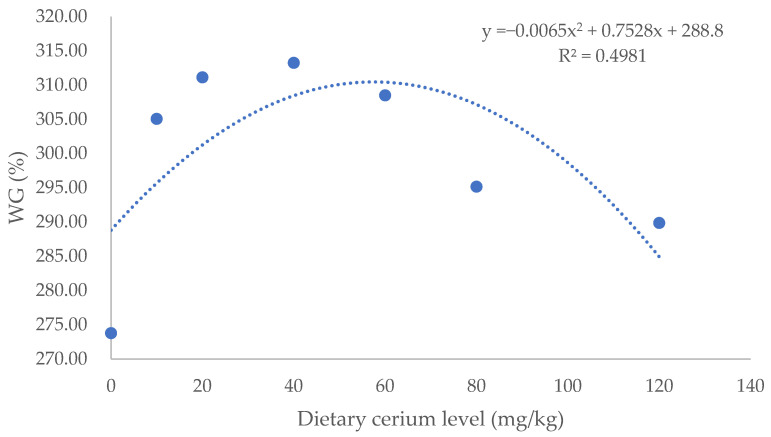
A quadratic regression curve was fitted with the dietary cerium (IV) inclusion as the independent variable X (mg/kg), and WG (%) as the dependent variable Y.

**Figure 2:**
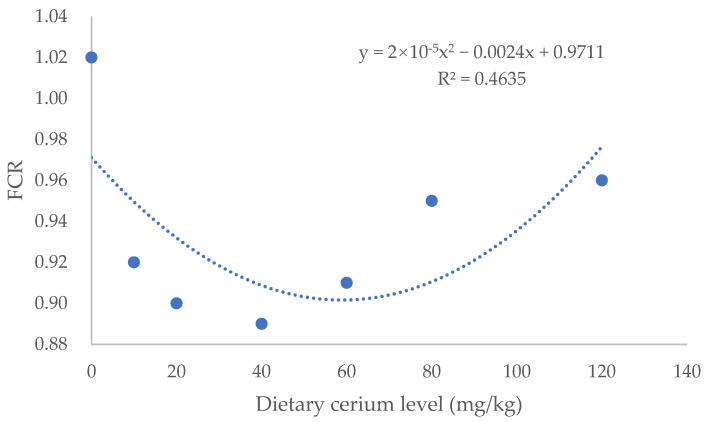
A quadratic regression curve was fitted with the dietary cerium (IV) inclusion as the independent variable X (mg/kg), and the FCR as the dependent variable Y.

After 56 days of feeding, all fish presented good performance without mortality being recorded. In Table 2, dietary cerium significantly affected WG and the FCR of largemouth bass. The Ce40 group showed the highest WG and lowest FCR with 14.4% higher WG and 0.13 lower FCR than the control group (*p* < 0.05). There were no significant differences in SR, VSI, HSI and CF among treatments (*p* > 0.05) (Table 3). The regression analysis revealed that WG was greatest and the FCR was the lowest when dietary cerium addition was 57.9, and 60.0 mg/kg, respectively (Figure 1 and Figure 2).

### 3.2. Nutrient Retention and Whole-Body Composition

The addition of 40 mg/kg, or 60 mg/kg dietary cerium tended to promote protein retention (*p* < 0.10), although the increase was not significant (*p* > 0.05). No significant differences were detected in lipid retention and the moisture, crude protein, crude fat, and crude ash contents of whole fish among all groups (*p* > 0.05) (Table 4).

### 3.3. Serum Biochemical Indicators

In antioxidant capacity, SOD activity was significantly increased, while MDA concentration was significantly decreased (*p* < 0.05) in the Ce60, Ce80 and Ce120 groups compared with the control group. The Ce120 group also presented a significantly higher T-AOC than the control (*p* < 0.05).

In non-specific immunity, the total protein concentration, the ACP and AKP activities in the Ce60 group, as well as the T-NOS activity in the Ce80 group were significantly higher than those in the control (*p* < 0.05). There were no statistically significant differences (*p* > 0.05) in the CAT and LZM activities and IgM level among all groups (Table 5).

### 3.4. Intestinal Digestive Enzyme Activity and Tissue Morphology

**Figure 3:**
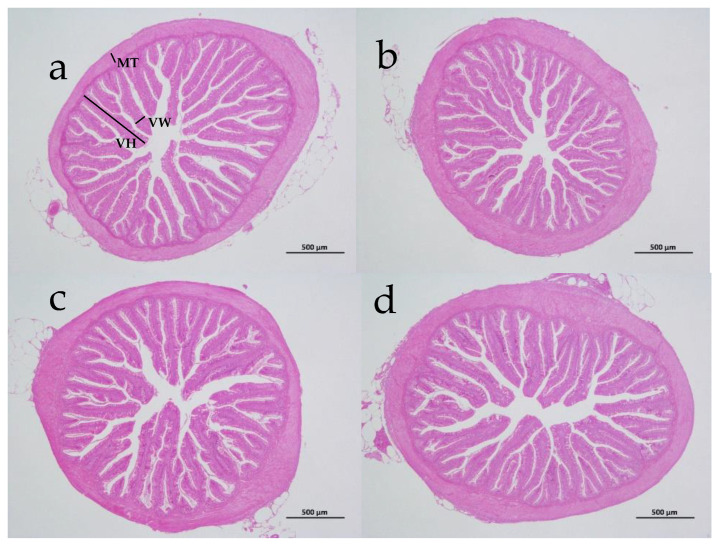
The intestinal tissue morphology of largemouth bass fed diets containing various levels of dietary cerium (40×). The letters (**a**–**d**) represent the control, Ce10, Ce40, and Ce120 groups, respectively (MT, muscular thickness; VH, villus height; VW, villus width).

The intestinal protease activity in the Ce20 and Ce40 groups, and the amylase activity in the Ce40 group were significantly increased compared with the control group (*p* < 0.05) (Table 6). As shown in Table 7 and Figure 3, there were no differences in the intestinal villi height, width and muscularis propria thickness among groups (*p* > 0.05).

### 3.5. Intestinal Microbiota

**Figure 4:**
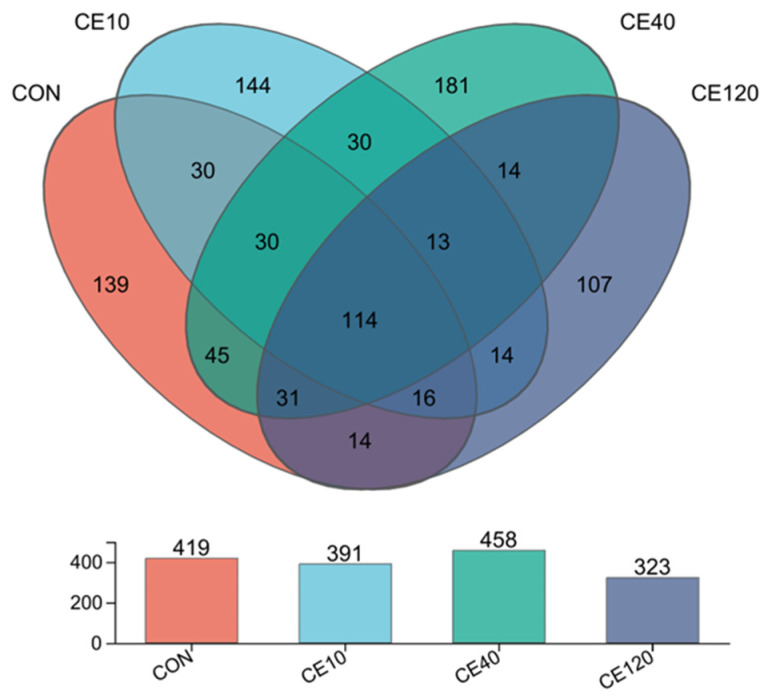
Venn diagram of OTUs of largemouth bass.

At the OTU (Operational Taxonomic Unit) level, the Ce40 group presented significantly higher Chao, Sobs and Ace indices than the Ce120 group (Table 8). The Venn diagrams showed that the OTUs in each group ranged from 323 to 419. The common OTUs in the CON, Ce10, Ce40, and Ce120 groups were 114, and the specific OTUs were 139, 144, 181, and 107 for the four groups, respectively (Figure 4).

**Figure 5:**
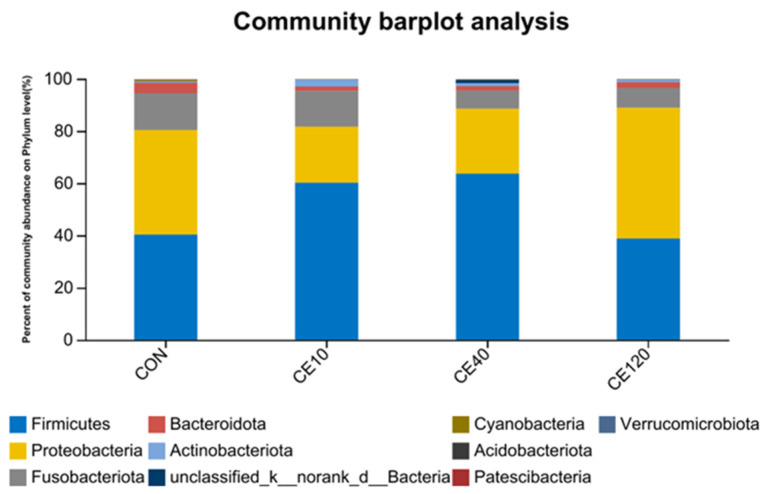
Relative abundance of intestinal microbiota of largemouth bass at th phylum level.

The dominant phyla were the Firmicutes, Fusobacteriota, and Proteobacteria, and the total abundance proportions of the three phyla were 94.45%, 95.33%, 95.46%, and 96.50% in the CON, Ce10, Ce40, and Ce120 groups, respectively. The abundance of Firmicutes was increased and the abundance of Proteobacteria was decreased by the addition of 10 mg/kg and 40 mg/kg dietary cerium (Ce10 and Ce40 groups) (Figure 5).

**Figure 6:**
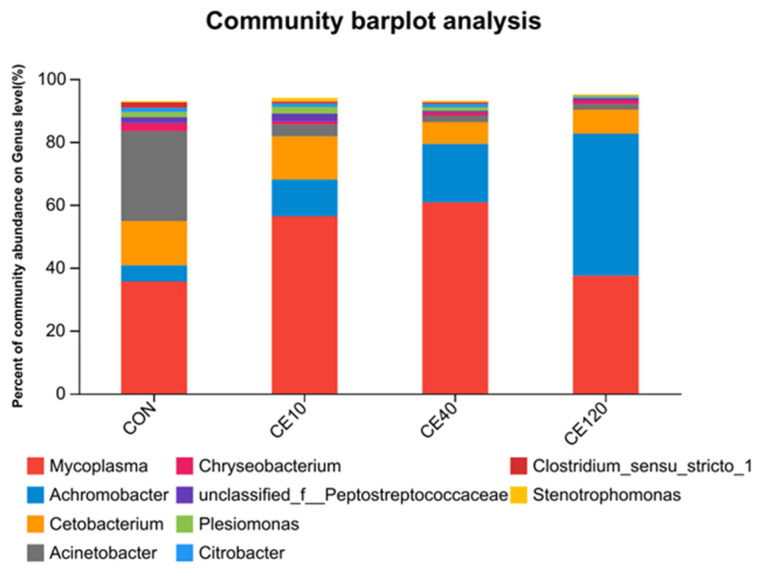
Relative abundance of intestinal microbiota of largemouth bass at the genus level.

The dominant genus in the control group was *Mycoplasma* (35.58%), followed by *Cetobacterium* (14.09%), and *Acinetobacter* (7.87%). The dominant genus in the Ce10, Ce40, and Ce120 groups were *Mycoplasma* with abundances of 56.38%, 60.76%, and 37.46%, and the other dominant genus was *Cetobacterium* (13.70%, 6.93%, and 7.64%) and *Achromobacter* (11.66%, 18.58%, and 45.13%). Compared with the control group, the abundance of *Cetobacterium* decreased by 50.82% and the abundance of *Mycoplasma* and *Achromobacter* increased by 70.77% and 136.09% in the Ce40 group (Figure 6).

## 4. Discussion

### 4.1. Growth Performance

The element cerium has two valences, 3^+^ and 4^+^, and the cerium in cerium ammonium nitrate [Ce (NH_4_)_2_(NO_3_)_6_] is 4^+^. Cerium has shown various physiological functions, and it can affect the organism’s metabolism [15]. Qin et al. [8] reported that dietary addition of 0.8 mg/kg of cerium oxide nanoparticles enhanced the WG of Chinese mitten crabs from 108.9% to 145.3% and reduced the FCR by 0.44. In common carp [4], the addition of 200–300 mg/kg of mixed rare earth nitrate (containing cerium nitrate 14.44%) also exhibited the same trend. The growth-promoting effect of rare earth elements is concerned with the improvement in gastrointestinal digestive enzyme activity, the regulation of intestinal microbial communities, the increase in nutrient utilization, the regulation of hormone secretion, etc. [16,17,18,19]. It is worth noting that high concentrations of rare earth elements may inhibit or slow down growth. For example, dietary addition of 600 mg/kg of cerium–chitosan complex (containing 90 mg/kg of Ce) increased the specific growth rate (SGR) of sea cucumber (*Apostichopus japonicas*) from 0.90%/d to1.21%/d, but the addition of 1200 mg/kg decreased the SGR to 0.96%/d [20]. In the present study, the addition of 40 mg/kg cerium significantly increased WG and decreased the FCR, but higher inclusion did not further increase WG and decrease the FCR. Based on the second-order polynomial regression analysis of WG or the FCR, the appropriate inclusion level of dietary cerium for juvenile largemouth bass was estimated to be 57.9 and 60.0 mg/kg, respectively.

### 4.2. Anti-Oxidative Capacity and Non-Specific Immune Indicators

Balanced homeostasis between reactive oxygen species and antioxidant capacity is essential for fish health. Enzymes such as SOD and CAT are important components of the organism’s antioxidant defense [21]. Rare earth elements can affect the oxidative state and antioxidant capacity of animals. In Chinese mitten crabs, dietary cerium oxide nanoparticles increased hemolymph SOD activity and decreased MDA content under ammonia stress [8]. In turbot, serum SOD activity was also increased by the addition of 240 mg/kg of rare earth nitrate (containing 20% rare earth) [2]. In this study, a high inclusion of dietary cerium (≥60 mg/kg) significantly increased serum SOD activity and decreased serum MDA concentration, and the serum T-AOC reached the highest value when dietary cerium was added at 120 mg/kg. This may be related to the continuous stimulation of high doses of dietary cerium. However, the increasing antioxidant capacity does not always mean a beneficial effect on the organism. For example, Azomite is a hydrated aluminosilicate mineral with plenty of rare earth, and the antioxidant capacity of largemouth bass was increased by the increasing addition of Azomite (1.0–6.0 g/kg), but growth showed the best performance when Azomite addition was 2 g/kg [22].

Non-specific immunity, also known as innate immunity, is an innately organic barrier in animals to prevent the invasion of pathogens, which includes the enzyme system (AKP, ACP, etc.) and complement system (C3, etc.) [13]. AKP is a regulatory enzyme involved in physiological functions in the organism, and ACP is a classical lysosomal enzyme that plays a key role in resisting pathogens [23]. C3 can activate and regulate the immune system through a variety of pathways such as the classical pathway, the alternative pathway, and the lectin pathway [24,25]. NOS can be induced by pathogens and cytokines to produce NO free radicals, which can increase the immune function of phagocytes. Rare earth elements possess similar effects to immunostimulants. Cui et al. [2] reported that the inclusion of 200–300 mg/kg of rare earth nitrate effectively activated the phagocytosis function of turbot cells to enhance non-specific immunity and resistance to *Echwardsiellatarda*. In Chinese mitten crabs, the supplementation of 0.8 mg/kg cerium oxide nanoparticles increased the blood cell count and serum activities of LZM, AKP, and ACP and enhanced survival in ammonia stress and immunity against bacterial infection [8]. Similarly, dietary addition of 600 mg/kg cerium–chitosan chelate also significantly increased phagocytic activity and the AKP and ACP activities of sea cucumber [20]. In the present study, when dietary cerium addition reached 60 mg/kg, AKP and ACP activities, as well as the total protein content, increased significantly, but higher inclusion (≥80 mg/kg) tended to decrease AKP and ACP activities. The T-NOS activity of the Ce80 group was significantly higher than that of the other groups. It is noteworthy that serum C3 content reached the highest value when the addition was 40 mg/kg, but the 120 mg/kg dietary cerium group showed significantly lower C3 content than the Ce40 group. The present results indicate that low and medium doses of dietary cerium additions promote the non-specific immunity of largemouth bass, while high addition might suppress some key immune-related factors. Similar findings have also been observed in Chinese mitten crabs [8], largemouth bass [25], and Gibel carp [6], which may be concerned with the immunosuppressive effect induced by high doses of rare earth elements [26].

### 4.3. Intestinal Digestive Enzyme Activity and Tissue Morphology

The intestine is an important place for the digestion and absorption of nutrients, and digestive enzymes in the intestine, such as protease, amylase, lipase, etc., play crucial roles in this process, while intestinal tissue morphology influences the contact area of intestinal mucosa and the chyme, directly affecting the efficiency of digestion and absorption. In the present study, foregut trypsin activity in the Ce20 and Ce40 groups and amylase activity in the Ce40 group were significantly higher than those in the control group, which may be one of the reasons for the growth improvement in largemouth bass. In common carp, the activities of intestinal lipase, trypsin and chymotrypsin, as well as growth, were also improved by rare earth nitrate addition (200–300 mg/kg), but high concentrations of rare earth nitrate inhibited the activities of the above enzymes [4]. In the present study, a decreasing trend of digestive enzyme activities was also observed in the high dietary cerium concentration group, which may be attributed to the inhibition of digestive enzyme activities by excessive rare earth content.

In intestinal tissue morphology, the density of mucus cells in the midgut and hindgut of common carp was significantly increased by cerium nitrate addition (20, 42, and 65 mg/kg) [27]. No significant difference in foregut tissue structure was observed among the groups in this study. Maybe dietary cerium just simulates digestive enzyme secretion but does not promote the development of intestine tissue morphology. Enhanced nutrient absorption may result from the up-regulation of micro-level functions rather than from the changes in macroscopic morphology. In addition, physiological and biochemical responses usually occur faster than tissue remodeling, and the sampling time points in this study may precisely capture this phase of functional activation.

### 4.4. Intestinal Microbiota

Fish and the microorganisms in their intestine are interdependent, and the formation of a unique microbial community structure is related to the fish’s food and water environment [28]. In this study, the dominant phyla in largemouth bass are Firmicutes, Fusobacteriota, and Proteobacteria, which is consistent with previous results in largemouth bass [29,30]. Generally, Proteobacteria are considered harmful bacteria in the intestine of fish [31]. Firmicute can secrete a variety of enzymes in metabolism to facilitate digestion and absorption of food [32]. In the present study, the abundance of Firmicutes significantly increased and the abundance of Proteobacteria significantly decreased in the Ce10 and Ce40 groups when compared with the control group. At the genus level, the abundance of *Acinetobacter* decreased sharply with the increasing dietary cerium level, while the abundance of *Achromobacter* gradually increased. It is noteworthy that the abundance of *Mycoplasma* increased and then decreased with the increase in dietary cerium concentration in the feed. In microbiota abundance, the Ce40 group had the highest endemic species number and the highest species diversity, while the Ce120 group had the lowest values, which may be attributed to the growth-promoting effect of dietary cerium at moderate concentrations and the inhibition of bacterial growth at high concentrations [33]. In in vitro experiments, cerium ion concentrations below 350 μg/mL stimulated the growth of *Escherichia coli*, while high concentrations (>400 μg/mL) may have had an inhibitory effect [34]. Previous studies have shown that some microorganisms in the intestines of fish appear to promote growth of the host [35,36]. The growth performance of largemouth bass in this study also showed a trend of increasing and then decreasing with the increase in dietary cerium concentration, and such results correlated with the changes in microorganisms, which needs to be further investigated.

## 5. Conclusions

In this study, dietary addition of dietary cerium promoted the growth, intestinal digestive enzyme activity, serum antioxidant and immune capacity, and positive modulation of the intestinal microflora community of juvenile largemouth bass. Based on the second-order polynomial regression analysis of WG or the FCR, the appropriate inclusion level of dietary cerium was estimated to be 57.9 and 60.0 mg/kg, respectively.

## Figures and Tables

**Table 1:** Ingredients and proximate composition of experimental diets (air-dry basis, g/kg).

Ingredients ^a^	CON	Ce10	Ce20	Ce40	Ce60	Ce80	Ce120
Fish meal	350.0	350.0	350.0	350.0	350.0	350.0	350.0
Soybean meal	80.0	80.0	80.0	80.0	80.0	80.0	80.0
Soy protein concentrate	110.0	110.0	110.0	110.0	110.0	110.0	110.0
Cottonseed protein concentrate	50.0	50.0	50.0	50.0	50.0	50.0	50.0
Wheat flour	125.00	124.96	124.92	124.84	124.76	124.68	124.52
Pork meal	80.0	80.0	80.0	80.0	80.0	80.0	80.0
Other ingredients ^b^	165.0	165.0	165.0	165.0	165.0	165.0	165.0
Vitamin premix ^c^	10.0	10.0	10.0	10.0	10.0	10.0	10.0
Mineral premix	30.0	30.0	30.0	30.0	30.0	30.0	30.0
Cerium (Ce (NH_4_)_2_(NO_3_)_6_)	0.00	0.04	0.08	0.16	0.24	0.32	0.48
Total	1000.0	1000.0	1000.0	1000.0	1000.0	1000.0	1000.0
Proximate composition							
Crude protein	483.7	481.9	482.2	482.1	481.7	486.2	482.5
Crude lipid	129.6	128.5	129.3	130.6	130.4	129.7	131.4
Ash	92.2	96.4	98.5	89.6	98.6	95.3	97.1
Moisture	113.5	107.4	105.3	112.2	115.9	114.6	108.5

^a^ The crude protein contents of fish meal, soybean meal, soy protein concentrate, cottonseed meal, pork meal, flour, and corn gluten meal were 672, 442, 654, 600, 740, 144, and 752 g/kg, respectively. ^b^ Corn gluten meal (40.0 g/kg), squid paste (40.0 g/kg), fish oil (25.0 g/kg), soybean oil (25.0 g/kg), and soybean lecithin (25.0 g/kg). ^c^ Vitamin and mineral composition referred to the description of Lin et al. [10].

**Table 2:** Effects of dietary cerium (IV) on growth performance of largemouth bass.

Items	CON	Ce10	Ce20	Ce40	Ce60	Ce80	Ce120
IBW/g	16.91 ± 0.02	16.88 ± 0.04	16.88 ± 0.04	16.90 ± 0.09	16.89 ± 0.02	16.89 ± 0.02	16.88 ± 0.02
FBW/g	63.54 ± 2.94 ^a^	68.86 ± 2.79 ^ab^	69.89 ± 2.47 ^ab^	70.25 ± 3.91 ^b^	69.44 ± 3.15 ^ab^	67.17 ± 2.77 ^ab^	66.27 ± 2.52 ^ab^
WG/%	273.75 ± 17.27 ^a^	305.05 ± 16.41 ^ab^	311.11 ± 14.51 ^ab^	313.22 ± 23.01 ^b^	308.47 ± 18.52 ^ab^	295.14 ± 16.27 ^ab^	289.85 ± 14.81 ^ab^
FCR	1.02 ± 0.06 ^a^	0.92 ± 0.05 ^ab^	0.90 ± 0.04 ^ab^	0.89 ± 0.07 ^b^	0.91 ± 0.05 ^ab^	0.95 ± 0.05 ^ab^	0.96 ± 0.05 ^ab^
SR/%	100.0	100.0	100.0	100.0	100.0	100.0	100.0

Note: Values are mean ± standard deviation (*n* = 3 cages per treatment; total 3/treatment). Values in the same row with different superscript letters are significantly different (*p* < 0.05).

**Table 3:** Effects of dietary cerium (IV) on body morphometric indices of largemouth bass.

Items	CON	Ce10	Ce20	Ce40	Ce60	Ce80	Ce120
VSI/%	5.75± 0.35	5.79 ± 0.53	5.62 ± 0.63	5.72 ± 0.46	5.68 ± 0.50	5.83 ± 0.27	5.78 ± 0.47
HSI/%	1.04 ± 0.22	1.09 ± 0.20	1.02 ± 0.09	1.07 ± 0.13	1.04 ± 0.12	1.06 ± 0.13	1.04 ± 0.19
CF (g/cm^−3^)	2.03 ± 0.08	2.07 ± 0.10	2.05 ± 0.16	2.06 ± 0.07	2.02 ± 0.15	2.01 ± 0.13	2.05 ± 0.06

Note: Values are mean ± standard deviation (*n* = 3 fish per cage; total 9/treatment).

**Table 4:** Effects of dietary cerium (IV) on nutrient retention and whole-body composition of largemouth bass.

Items	CON	Ce10	Ce20	Ce40	Ce60	Ce80	Ce120
Protein retention/%	37.20 ± 3.02	40.08 ± 2.90	40.15 ± 1.90	40.63 ± 2.82	40.96 ± 2.84	38.21 ± 1.78	37.54 ± 1.63
Lipid retention/%	51.60 ± 1.52	49.96 ± 0.57	49.38 ± 0.67	49.02 ± 4.48	50.07 ± 2.76	50.83 ± 8.77	50.09 ± 10.17
Moisture/%	71.12 ± 1.17	71.42 ± 0.23	70.95 ± 0.20	71.66 ± 0.43	70.62 ± 0.72	71.05 ± 0.39	70.73 ± 0.34
Crude protein/%	17.20 ± 0.13	17.37 ± 0.27	17.15 ± 0.02	17.28 ± 0.12	17.51 ± 0.53	17.18 ± 0.13	17.19 ± 0.16
Crude lipid/%	5.79 ± 0.80	5.65 ± 1.59	5.63 ± 0.19	5.44 ± 0.09	5.61 ± 0.50	5.86 ± 1.67	5.77 ± 1.05
Crude ash/%	4.52 ± 0.93	4.29 ± 0.47	4.43 ± 0.24	4.27 ± 0.49	4.15 ± 0.20	4.52 ± 0.12	4.37 ± 0.05

Note: Values are mean ± standard deviation (*n* = 3 fish per cage; total 9/treatment).

**Table 5:** Effects of dietary cerium (IV) on serum biochemical indicators of largemouth bass.

Items	CON	Ce10	Ce20	Ce40	Ce60	Ce80	Ce120
SOD (u/mL)	222.22 ± 27.35 ^a^	228.88 ± 17.93 ^ab^	259.2 ± 16.17 ^ab^	229.27 ± 25.88 ^ab^	275.53 ± 23.96 ^b^	270.99 ± 3.54 ^b^	273.49 ± 9.62 ^b^
CAT(U/mL)	9.96 ± 1.69	10.73 ± 0.22	10.61 ± 0.45	10.52 ± 2.36	10.30 ± 0.83	11.68 ± 0.16	10.79 ± 2.94
T-AOC (U/ml)	42.92 ± 1.98 ^a^	45.84 ± 3.20 ^ab^	48.10 ± 1.40 ^ab^	44.03 ± 3.31 ^ab^	49.95 ± 4.71 ^ab^	49.69 ± 3.61 ^ab^	50.94 ± 2.97 ^b^
MDA (nmol/mL)	4.47 ± 0.06 ^a^	4.39 ± 0.26 ^ab^	4.34 ± 0.63 ^ab^	4.20 ± 0.24 ^ab^	3.58 ± 0.06 ^bc^	3.65 ± 0.44 ^bc^	3.32 ± 0.31 ^c^
TP (gprot/L)	33.92 ± 0.79 ^a^	35.55 ± 0.54 ^ab^	35.79 ± 1.50 ^ab^	36.45 ± 1.10 ^ab^	36.93 ± 0.58 ^b^	35.61 ± 1.81 ^ab^	36.33 ± 2.26 ^ab^
AKP (U/mL))	45.37 ± 4.82 ^a^	54.87 ± 7.63 ^ab^	49.46 ± 7.90 ^ab^	56.82 ± 9.06 ^ab^	62.27 ± 9.06 ^b^	54.96 ± 3.39 ^ab^	44.56 ± 3.28 ^a^
ACP (U/mL)	0.22 ± 0.01 ^a^	0.23 ± 0.02 ^ab^	0.24 ± 0.03 ^ab^	0.24 ± 0.02 ^ab^	0.26 ± 0.02 ^b^	0.24 ± 0.01 ^ab^	0.22 ± 0.01 ^ab^
LZM (μg/mL)	7.66 ± 1.37	7.88 ± 1.37	7.66 ± 1.09	7.55 ± 1.19	7.55 ± 0.85	7.60 ± 0.72	7.60 ± 1.19
T-NOS (U/mL)	34.4 ± 2.00 ^a^	36.67 ± 2.00 ^a^	35.39 ± 1.80 ^a^	36.06 ± 0.70 ^a^	38.30 ± 0.30 ^a^	43.68 ± 2.10 ^b^	36.31 ± 3.30 ^a^
IgM (μg/mL)	5.70 ± 0.73	5.58 ± 1.56	6.21 ± 0.18	5.17 ± 0.73	5.20 ± 0.38	6.03 ± 0.51	5.64 ± 0.15
C3(μg/mL)	20.96 ± 3.68 ^ab^	21.57 ± 2.71 ^ab^	20.87 ± 1.72 ^ab^	24.61 ± 2.46 ^a^	22.39 ± 2.52 ^ab^	21.52 ± 1.29 ^ab^	17.27 ± 1.29 ^b^

Note: Values are mean ± standard deviation (*n* = 3 fish per cage; total 9/treatment). Values in the same row with different superscript letters are significantly different (*p* < 0.05).

**Table 6:** Effects of dietary cerium (IV) on intestinal digestive enzyme activities of largemouth bass.

Items	CON	Ce10	Ce20	Ce40	Ce60	Ce80	Ce120
Protease/(U/mgprot)	4861.0 ± 455.7 ^ab^	5347.5 ± 58.7 ^bc^	5640.5 ± 35.0 ^c^	5718.8 ± 289.1 ^c^	5168.3 ± 383.9 ^bc^	4528.0 ± 140.9 ^ab^	4495.5 ± 35.1 ^ab^
Amylase/(U/mgprot)	0.29 ± 0.02 ^a^	0.34 ± 0.00 ^ab^	0.35 ± 0.00 ^ab^	0.40 ± 0.02 ^b^	0.36 ± 0.02 ^ab^	0.34 ± 0.00 ^a^	0.32 ± 0.07 ^a^

Note: Values are mean ± standard deviation (*n* = 3 fish per cage; total 9/treatment). Values in the same row with different superscript letters are significantly different (*p* < 0.05).

**Table 7:** Effects of dietary cerium (IV) on intestinal tissue morphology of largemouth bass.

Items	CON	Ce10	Ce40	Ce120
Villi height/μm	855.0 ± 17.4	852.7 ± 20.7	877.3 ± 8.5	878.7 ± 39.8
Villi width/μm	91.3 ± 0.5	91.8 ± 2.6	94.0 ± 3.0	94.0 ± 2.4
Muscularis propria thickness/μm	132.3 ± 3.1	135.3 ± 2.5	134.3 ± 1.5	135.7 ± 0.6

Note: Values are mean ± standard deviation (*n* = 3 fish per cage; total 9/treatment).

**Table 8:** Diversity index on OTU level of intestinal microbiota of largemouth bass.

Items	CON	Ce10	Ce40	Ce120
Chao	144.3 ± 35.7 ^ab^	135.8 ± 13.4 ^ab^	168.1 ± 42.7 ^b^	111.0 ± 16.9 ^a^
Sobs	118.3 ± 30.3 ^ab^	115.0 ± 8.5 ^ab^	155.0 ± 38.5 ^b^	104.5 ± 12.9 ^a^
Ace	144.5 ± 32.5 ^ab^	129.8 ± 10.4 ^ab^	166.7 ± 41.3 ^b^	109.7 ± 15.8 ^a^
Coverage	0.999	0.999	0.999	0.999

Note: Values are mean ± standard deviation (*n* = 4 fish pooled as 2 samples per cage; total 6/treatment). Values in the same row with different superscript letters are significantly different (*p* < 0.05).

## Data Availability

Data are contained within the article.
